# Quantifying the Ecosystem Services of Soda Saline-Alkali Grasslands in Western Jilin Province, NE China

**DOI:** 10.3390/ijerph19084760

**Published:** 2022-04-14

**Authors:** Lei Chang, Zhibo Zhao, Lixin Jiang, Yuefen Li

**Affiliations:** 1 College of Earth Sciences, Jilin University, Changchun 130061, China; changlei2217@mails.jlu.edu.cn (L.C.); jianglx18@mails.jlu.edu.cn (L.J.); 2 Faculty of Built Environment, University of Malaya, Kuala Lumpur 50603, Malaysia; 17220374@siswa.um.edu.my

**Keywords:** land ecology, ecosystem services, InVEST model, saline-alkali grassland, synergy, trade-off

## Abstract

This study aimed to quantitatively describe the ecosystem services of soda saline-alkali grasslands based on literature research, the InVEST model, a transition matrix, and Spearman’s correlation analysis. The chosen methodology could provide insight into the relationships between different services to provide empirical evidence for decision-making concerning the protection and restoration of saline-alkali grasslands. The research provided several insights into the ecological situation in western Jilin Province. First, the area of saline-alkali grassland in western Jilin Province had noticeably decreased from 1990 to 2018. Moreover, the threat of grassland degradation in western Jilin Province has increased year by year, and has become the main problem facing the ecological environment of this region. Second, the results demonstrated how the amount of grassland area, and coverage, are intricately linked to the provided ecosystem services, and maintaining the stability of ecosystem services is the basis for future efforts to increase grassland area and coverage. A trade-off relationship exists between water supply services and other ecosystem services, which indirectly confirms a climatic cause for grassland salinization in western Jilin Province. The analyses identified various types of grassland ecosystem service hotspots, but the share of hotspots representing all four assessed ecosystem services was small; this indicates that the grassland ecosystem of western Jilin Province is of generally poor quality. In conclusion, increasing grassland salinization has reduced vegetation coverage, which leads to the degradation of the grassland ecosystem and, in turn, affects the relationships between various ecosystem services.

## 1. Introduction

Given the extent of ecosystem degradation resulting from environmental changes and human activity [1,2], restoration efforts have increasingly focused on biodiversity and ecosystem services [3,4]. Grassland, which is widely distributed around the globe, is an important terrestrial ecosystem due to a wide range of ecosystem services such as climate regulation, wind prevention and sand fixation, water conservation, biodiversity protection, and carbon sequestration [5,6,7,8,9]. Grassland is also an important ecosystem and natural resource in China, covering an area of approximately 4 million km^2^, which accounts for more than 40% of the country’s land area and represents a major contributor to net ecosystem productivity [10]. The grasslands in the western part of Jilin Province are an important animal husbandry production base [11], as well as an important economic pillar of the local area. However, grasslands are under severe threat from ongoing degradation, which undermines their capacity to support biodiversity, ecosystem services, and human well-being [5]. The grasslands of western Jilin Province are located in the saline-alkali soil area of the Songnen Plain, which is one of the three saline-alkali areas in the world [11,12,13]. Recently, grassland salinization in the region has become increasingly serious due to global climate change and human factors. As a result, grassland ecosystem productivity has significantly decreased, and there has also been a reduction in soil carbon regression and carbon sequestration ability [14,15]. Regarding this ecological degradation and general unsustainability, the Chinese government considers the ecosystem of mountains, rivers, forests, fields, lakes, and grassland to be an inseparable ecosystem and community of life, with the systemic management of these biotopes essential to ecological restoration [16,17,18]. Therefore, the protection of grassland ecosystems is of great significance, and enhancing the ecological service function of grasslands will ensure ecological security and promote sustainable development.

Ecosystem services has become a prominent concept in international policy and research agendas [19] and has gained traction for its ability to link societal benefits to the underlying ecology and functioning of ecosystems [20]. Ecosystem services are the natural utilities formed by ecosystems and ecological processes that require human maintenance to survive and develop [21,22]. Complex interrelationships exist between different ecosystem services, and during the process of change, these mainly manifest as trade-offs and synergies of mutual gain. These interrelationships can be explained by the diversity of ecosystem services, uneven spatial distribution, and the selectivity of human use [23,24,25,26,27]. According to previous research in ecosystem services, these trade-offs can be quantified through four approaches: statistical methods; spatial analysis; scenario simulation; and ecosystem service mobility analysis [28]. Researchers have applied these methods to investigate grassland water conservation, windbreak and sand-fixing, and culture based on landscape patterns [24,29]. Developments in ecosystem services research have resulted in numerous researchers using the InVEST model to study how land use changes impact ecosystem services [26,28,30]. Grassland ecosystem services are closely related to human well-being [31]. However, previous research on grassland ecosystem services has mostly applied a certain model or method to measure changes in one or more grassland ecosystem services. Few scholars have studied the relationships between various grassland ecosystem services. Moreover, salinized grasslands, such as those in western Jilin Province have received limited research attention. The western part of Jilin Province, NE China, is the area most affected by salinization, and grassland is the main ecosystem in the area. The increasingly serious salinization will lead to a gradual decrease in the function of grassland, which will seriously threaten the ecological and economic development of the region. Therefore, investigating grassland ecosystem services in the study area, correctly recognizing the relationships between these services, and identifying ecosystem service hotspots [9,32] will be conducive to effectively classifying grassland ecosystem service types and promoting the sustainable development of the grassland ecosystem.

This paper focuses on the salinized grasslands of western Jilin Province and explores the relationships between different ecosystem services. This information, when combined with the spatiotemporal variation of grassland ecosystem service hotspots, can provide a basis for effective decision-making about grassland protection and restoration in western Jilin Province. The research results can also help improve the current understanding of the complex relationships between climate change, land use, and grassland ecosystem services and provide recommendations for future grassland use planning.

## 2. Materials and Methods

### 2.1. Research Area

**Figure 1:**
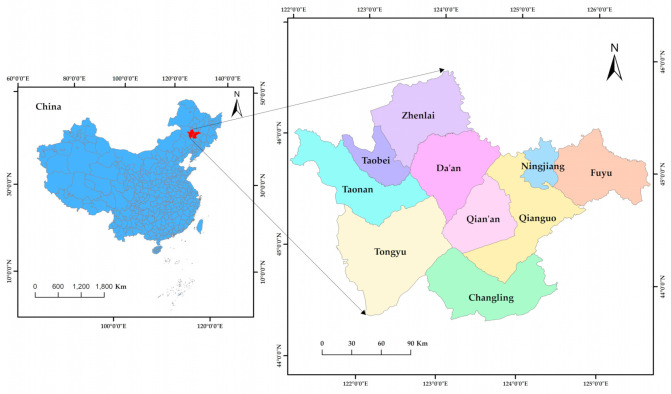
Map of Western Jilin Province.

Western Jilin Province is located in the east of Horqin Grassland and the southwest parts of the Songnen Plain, in the region of 43°59′ N–46°18′ N and 121°38′ E–126°11′ E. It has a temperate continental monsoon climate with average annual precipitation of 400–500 mm, and average evaporation of 1500–1900 mm, which is 3.5–4.75 times the precipitation. The main administrative areas include Songyuan City and Baicheng City. Of these, Songyuan City governs Ningjiang, Fuyu, Qian’an, Changling and Qianguo, whereas Baicheng governs Taobei, Taonan, Daan and Tongyu and Zhenlai (Figure 1). According to statistics from 2018, western Jilin province covers approximately 46,800 km^2^, with grassland accounting for 4700 km^2^. *Leymus chinensis* meadow is the main type of grassland in this area, with *Leymus chinensis* the main dominant species; this type of environment has a strong ability to adapt to drought and increasing salinity [33]. These grasslands are located in the saline-alkali soil area of northeast Songnen Plain and the eastern part of the farming-pastoral ecotone of northern China. These grasslands represent a typical fragile and degraded ecosystem.

### 2.2. Data Sources

Land use/cover change data (1990, 2000, 2010 and 2018) and elevation data were obtained from Resources and Environmental Science and Data Center (http://www.resdc.cn/, accessed on 15 June 2020) at the CAS Institute of Geographical Sciences and Natural Resources Research. The carbon density data were mainly obtained from related literature and InVEST model guides representing similar or identical regions. The precipitation data were obtained from the National Meteorological Data Center (https://data.cma.cn/, accessed on 7 December 2020). The soil data were obtained from the National Science and Technology Resource Sharing Service Platform (http://soil.geodata.cn, accessed on 7 December 2020). Other model parameters were obtained by simulation and debugging according to the InVEST operation method, or by referring to relevant policy documents, such as the InVEST model guidelines and ecological red line delineation guidelines.

### 2.3. Research Methods

#### 2.3.1. Research on Grassland Cover Change

Based on the land use transfer matrix, the land use change data from 1990, 2000, 2010 and 2018 for western Jilin province were analyzed to calculate the contribution rates of conversion-in and conversion-out. The transformation of grassland and other land types was analyzed. The land use transfer matrix includes information that reflects the dynamic process of the mutual transformation between different land types at the beginning and end of a certain period in a certain area. Information on the transfer-out and transfer-in of different land types was available for the end of the period. The spatial mapping function in ArcGIS software (Environmental Systems Research Institute, RedLands, America) was used to express these four types of LUCC (land use/cover change) data and analyze the spatial distribution characteristics of grassland from 1990 to 2018.

#### 2.3.2. Assessment of Grassland Ecosystem Services

Using InVEST 3.8.9 x86 software (Stanford University, San Francisco, CA, USA), we simulated four ecosystem services (carbon storage, soil conservation, water supply and habitat quality) across 1990–2018, with data coming from 1990, 2000, 2010 and 2018. When compared to other ecosystem service models, the InVEST model has certain obvious advantages and is widely used to measure ecosystem services [34,35,36]. The InVEST model can also provide a spatial visualization of ecosystem service functions. In comparison with other models, the InVEST model not only enables scenario prediction, but can also clearly show the correlation between ecosystem service functions.

Carbon storage was simulated by selecting the Carbon module in the InVEST model. This module adds the four basic carbon pools (aboveground C, underground C, soil C and dead organic matter) to obtain total carbon storage. Soil conservation was simulated by the SDR Sediment Delivery Ratio module of the InVEST model, i.e., soil conservation was the difference between potential soil erosion and actual soil erosion. The water supply was simulated by selecting the Water Yield module of the InVEST model, i.e., water supply is the amount of water when actual evapotranspiration is subtracted from precipitation. Habitat quality was simulated using the Habitat Quality module of the InVEST model, which generates results from land-use change data and information on biodiversity threats.

A detailed description of the InVEST model can be found in previous studies [37,38,39,40,41,42]. Therefore, a brief summary is presented here.

#### 2.3.3. Identification of Grassland Ecological Service Hotspots

On the basis of previous studies, this paper used statistical analysis to explore the trade-offs or synergies of ecosystem services [43,44]. Since the analyzed data did not demonstrate the characteristics of normal distribution, Spearman’s correlation analysis was used. If there are no repeated values in the data, and when the two variables are completely monotonically correlated, Spearman’s correlation coefficient is +1 or −1, i.e., either a synergistic or trade-off relationship, respectively. ArcGIS software was used to reclassify the grids based on which each ecosystem services showed values that exceeded the average from 1990 to 2018; ecological service hotspot maps of grassland with different coverage were obtained by superimposition.

## 3. Results

### 3.1. Analysis of Spatiotemporal Variation in the Characteristics of Grassland in Western Jilin Province

#### 3.1.1. Characteristics of Temporal Change of Grassland Cover

According to the land resources and their utilization attributes, a two-level classification system was adopted. According to the research objective, the adopted two-level classification system included grassland and unused land. Grassland was divided into high-cover, medium-cover and low-cover grassland, whereas unused land was divided into saline-alkali land and other unused land. In order to comprehensively understand the transformation of grassland in the study area, data for different land types in the study area were statistically analyzed, and the changes in area were determined. In particular, changes in the area of high-cover and low-cover grassland were particularly significant. The total area of high-cover grassland decreased from 527,254.25 hm^2^ in 1990 to 198,109.28 hm^2^ in 2018, whereas the area of low-cover grassland increased from 19,935.52 hm^2^ to 386,085.71 hm^2^.

In order to clarify the transformation in various land use types, this paper calculated the transformation of various land use types from 1990 to 2018 based on the contribution rates of conversion-in and conversion-out previously presented by Yu et al. [45] (Table 1).

As can be seen from Table 1, in the case of grassland, the contribution rate of conversion-out exceeded that of conversion-in during 1990–2018, with high-cover grassland showing the most serious degradation. From 2010 to 2018, the contribution rate of conversion-in for medium-cover grassland exceeded the contribution rate of conversion-out, but this type of grassland still showed a decreasing trend. For low-cover grassland, the contribution rate of conversion-out was lower than that of conversion-in throughout the analyzed period; however, because the total area of low-cover grassland was relatively small, the increase in this type of grassland was not enough to offset the degradation in high- and medium-cover grassland. In addition, from 1990 to 2018, the contribution rate of conversion-in for cultivated land was 44.61%, which was 31.30% higher than the corresponding conversion-out rate. Furthermore, the contribution rates of conversion-in for cultivated land and saline-alkali land were high between 1990–2000, with the main source of converted land being grassland.

#### 3.1.2. Spatial Variation in Grassland Cover

**Figure 2:**
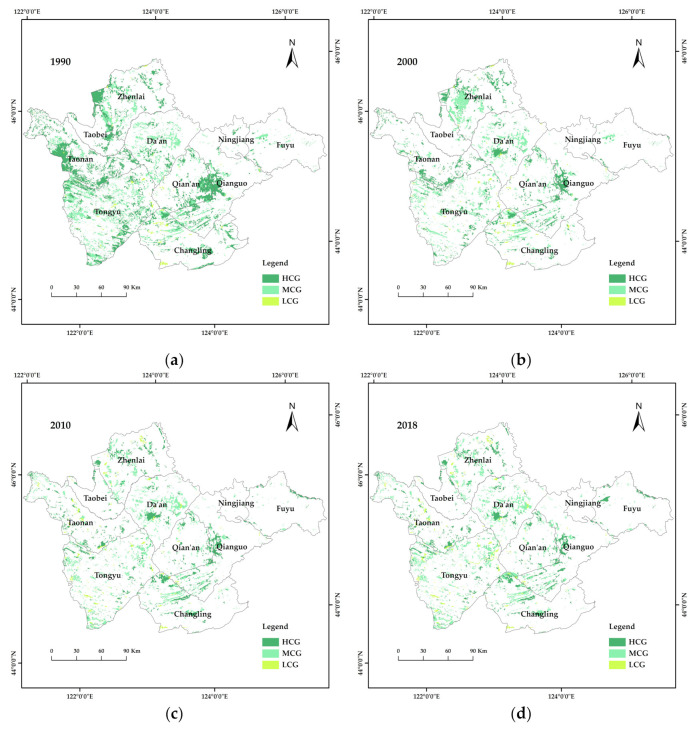
Grassland distribution in western Jilin Province from 1990 to 2018. HCG, High-cover grassland; MCG, Medium-cover grassland; LCG, Low-cover grassland. (**a**–**d**), spatial distribution of high, medium and low cover grassland in western Jilin province in 1990, 2000, 2010 and 2018, respectively.

From 1990 to 2018, grassland area decreased year by year, with the distribution gradually changing from local contiguous to scattered (Figure 2). In 1990, high-cover grassland was concentrated in the west of Zhenlai County, southwest of Taonan City and the junction of Qian’an County and Qianguo County. Medium-cover grassland was widely distributed, yet scattered, whereas there were small, and scattered, areas of low-cover grassland. The overall grassland area decreased significantly from 1990 to 2000, with high-cover grassland, which was mainly converted to arable land demonstrating the most serious degradation. In 2010, the area of grassland in Zhenlai County decreased sharply, and no concentrated area of high-cover grassland was visible, and high-cover grassland also disappeared from Taonan City and Tongyu County. The grassland distribution in 2018 was similar to what was observed in 2010.

### 3.2. Assessment of Grassland Ecosystem Services

#### 3.2.1. Evaluation of Carbon Storage Services

**Figure 3:**
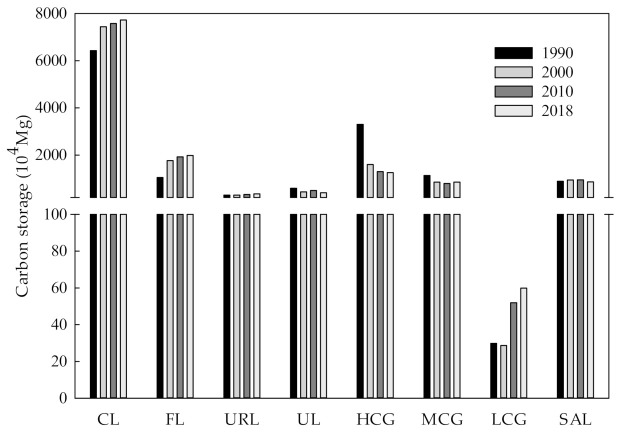
Total carbon storage of different land use types from 1990 to 2018. CL, Cultivated land; FL, Forest land; URL, Urban and rural construction land; UL, Unused land; HCG, High-cover grassland; MCG, Medium-cover grassland; LCG, Low-cover grassland; SAL, Saline-alkali land.

As can be seen from Figure 3, total carbon storage in the study area was 137.17 million Mg, 133.72 million Mg, 134.21 million Mg and 134.96 million Mg in 1990, 2000, 2010 and 2018, respectively; this is indicative of an overall downward trend. From 1990 to 2018, total carbon storage in the study area decreased by 2.22 million Mg (Mg = 10^6^ g, 1 Mg = 1t). From 1990 to 2018, the total carbon storage of grassland decreased year by year, with a total decrease of 22.96 million Mg. The carbon storage of high- and medium-cover grassland decreased by 20.48 million Mg and 2.78 million Mg, respectively. The total carbon storage of low-cover grassland increased slightly, with an increase of 300.02 million Mg. In 1990, the total carbon storage of high-cover grassland was two times greater than what was observed in 2000, which was consistent with the changes observed in high-cover grassland area. Although saline-alkali land has the lowest soil carbon density of the analyzed land types, saline-alkali land demonstrated greater carbon storage than low-cover grassland because the area of saline-alkali land greatly exceeded that of low-cover grassland.

**Figure 4:**
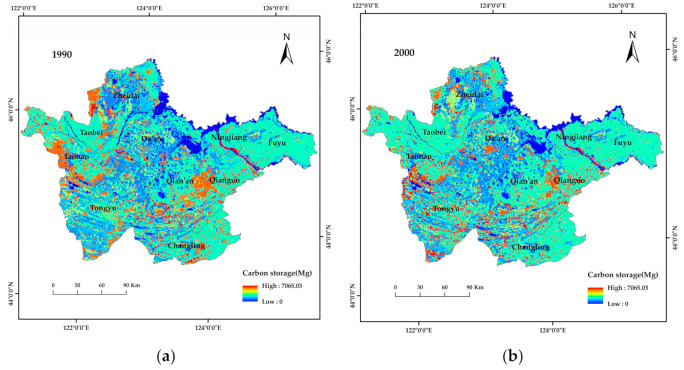

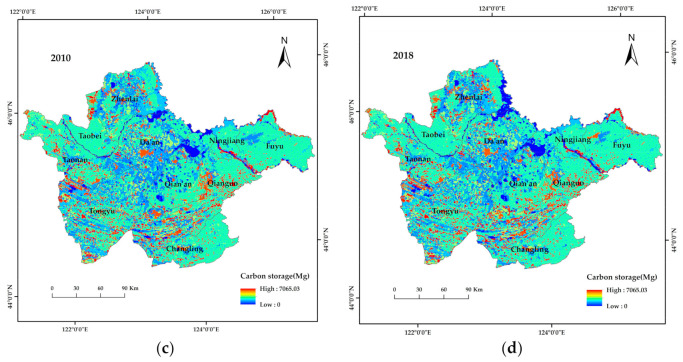
Spatial distribution of carbon storage in western Jilin Province from 1990 to 2018. (**a**–**d**), spatial distribution of carbon storage in western Jilin Province in 1990, 2000, 2010 and 2018, respectively.

Carbon storage can be divided into three grades: high value (4691.62–7065.03 Mg); median value (2345.81–4691.62 Mg); and low value (0–2345.81 Mg). As shown in Figure 4, from 1990 to 2018, the study area was dominated by middle- and low- value carbon storage areas, wheras the distribution of high value carbon storage areas showed a trend from local concentration to fragmentation. In 1990, three areas showed high carbon reserves, e.g., an area in the west of Zhenlai County, an area south of Taonan City, and an area close to the junction of Qianguo County and Qian’an County. This finding was consistent with the distribution of high-cover grassland in 1990. In 2000, the area of high- value carbon storage decreased sharply and showed fragmentation and dispersion. When the results illustrated in Figure 2 and Figure 4 are considered together, the spatial distribution of carbon storage in the study area showed a one-to-one correspondence with high-, middle-, and low-cover grassland distribution.

#### 3.2.2. Assessment of Soil Conservation Services

**Figure 5:**
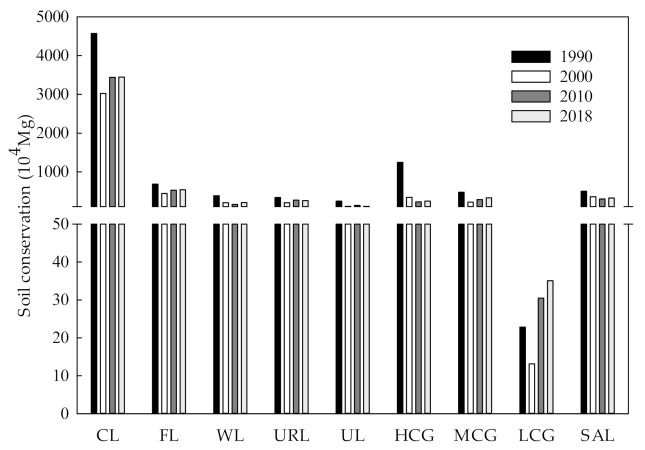
Total amount of soil conservation for different land use types from 1990 to 2018. CL, Cultivated land; FL, Forest land; WL, Water land; URL, Urban and rural construction land; UL, Unused land; HCG, High-cover grassland; MCG, Medium-cover grassland; LCG, Low-cover grassland; SAL, Saline-alkali land.

The total amount of soil conservation in the study area was 84.68 million Mg, 49.12 million Mg, 53.72 million Mg and 54.86 million Mg in 1990, 2000, 2010 and 2018, respectively; this is indicative of a decreasing trend from 1990 to 2010, and an increasing trend from 2010 to 2018 (Figure 5). Total soil conservation decreased from 1990 to 2018, with only low-cover grassland showing an increase of 219,300 Mg, or 53.58%, over the period. The soil conservation of high-cover grassland decreased by 10.01 million Mg, which was only exceeded by the decrease calculated for cultivated land. From 1990 to 2000, the total amount of soil conservation of cultivated land and high-cover grassland decreased by 15.50 million Mg and 9.05 million Mg, respectively.

**Figure 6:**
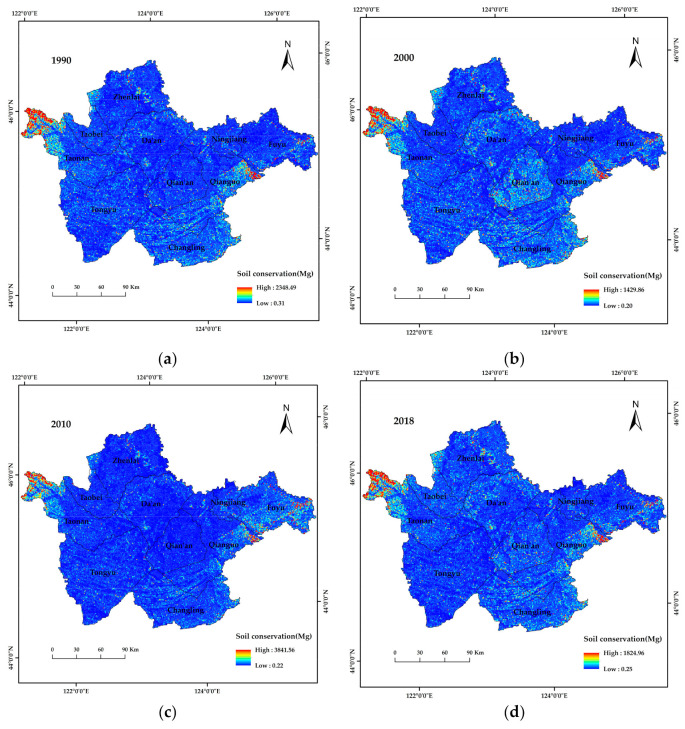
Spatial distribution of soil conservation in western Jilin Province from 1990 to 2018. (**a**–**d**), spatial distribution of soil conservation in western Jilin Province in 1990, 2000, 2010 and 2018, respectively.

According to Figure 6, soil conservation in the study area from 1990 to 2018 was predominantly in the low value range (<100 Mg), and only areas to the west of Taonan County, the east of Qianguo County and the east of Fuyu City showed high values (>1000 Mg) for soil conservation; these areas mainly included forest land and high-cover grassland, which indicates that forest land and high-cover grassland are valuable for soil conservation. In 2010, the amount of soil conservation in the entire study area increased, but the high value soil conservation previously observed in Changling County, Qian’an County and the Da’an range decreased significantly; the reason for this is that the three administrative regions showed relatively low values for the rainfall erosion factor, and according to the formula for soil conservation, a low rainfall factor value will directly decrease soil conservation. The spatial distribution of soil conservation in 2018 was similar to what was observed in 2000.

#### 3.2.3. Evaluation of Water Supply Services

**Figure 7:**
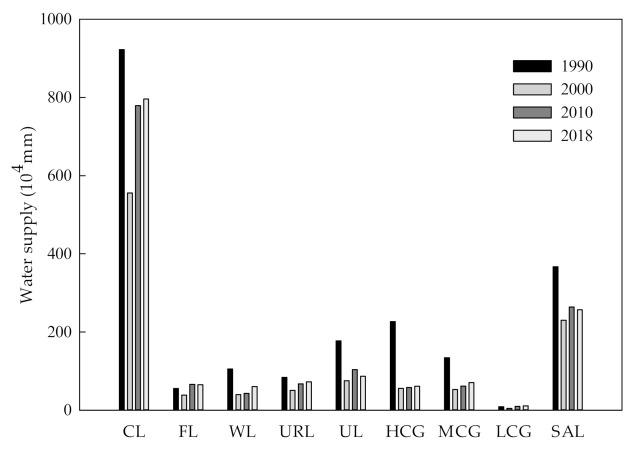
Total water supply of different land use types from 1990 to 2018. CL, Cultivated land; FL, Forest land; WL, Water land; URL, Urban and rural construction land; UL, Unused land; HCG, High-cover grassland; MCG, Medium-cover grassland; LCG, Low-cover grassland; SAL, Saline-alkali land.

The total amount of water supply in 1990, 2000, 2010 and 2018 was 3.67 million mm, 2.30 million mm, 2.64 million mm and 2.57 million mm, respectively, based on the InVEST model. From 1990 to 2018, the water supply in the study area showed a decreasing trend. The total amount of water conservation for high- and medium-cover grassland decreased from 1990–2000, and this trend could be mainly attributed to decrease in area of these land types. The variation in water supply for different land use types across the study period is illustrated in Figure 7.

**Figure 8:**
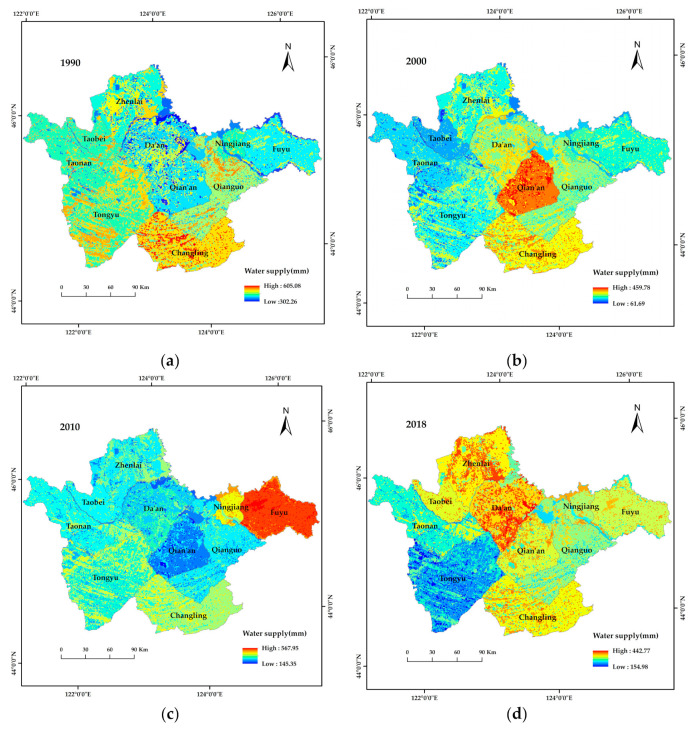
Spatial distribution of water supply in western Jilin Province from 1990 to 2018. (**a**–**d**), spatial distribution of water supply in western Jilin Province in 1990, 2000, 2010 and 2018, respectively.

Water supply is mainly affected by annual rainfall and annual potential evapotranspiration. During data collection, information on rainfall was organized by administrative region, so the spatial distribution of water supply from 1990 to 2018 mainly changed by administrative region. According to Figure 8, water supply in the study area ranged from 302.26–605.08 mm in 1990, with Changling County showing the greatest water supply. In 2000, the water supply ranged from 61.69–459.78 mm, with Qian’an County having greater water supply than Changling County. In 2010, the water supply ranged from 145.35–567.95 mm, with Fuyu City showing the greatest water supply. In 2018, the water supply ranged from 154.98–442.77 mm, with water supply greatest in Da’an City and Zhenlai County. The western parts of the study area demonstrated lower water supply capacity than the eastern parts of the study area.

#### 3.2.4. Habitat Quality Assessment

The average habitat quality indices in 1990, 2000, 2010 and 2018 were 0.5805, 0.5797, 0.5786 and 0.5786, respectively, based on the Habitat quality module of InVEST model. The habitat quality of a certain land-use type describes the relative sensitivity to various threats, the distance between threats, the distribution and density of each threat and the spatial location of the land use type. As shown in Table 2, the habitat quality indices of different land use types from 1990 to 2018 followed a similar trend to the annual average habitat quality index, which initially decreased and subsequently increased. The highest habitat quality index was noted in 1990 (0.5805). From 1990 to 2018, the annual average habitat quality index of grassland was the highest, i.e., 0.9814, 0.983 and 0.9851 for high-, medium- and low-cover grassland, respectively. Saline-alkali land had the lowest average habitat quality index (0.1996). The average habitat quality index results for different land types follow the order: low-cover grassland > medium-cover grassland > high-cover grassland > forest > cultivated land > water > saline-alkali land. The low-cover grassland shows the highest average value because it has a more scattered distribution and less contact with threats, whereas high-cover grassland accounts for a larger area, is relatively concentrated, and is in close contact with threats. From 1990 to 2018, only the habitat quality index of cultivated land increased by 0.0001. The habitat quality indices of other land types decreased, with high-cover grassland showing the most noticeable decrease (−0.0056).

**Figure 9:**
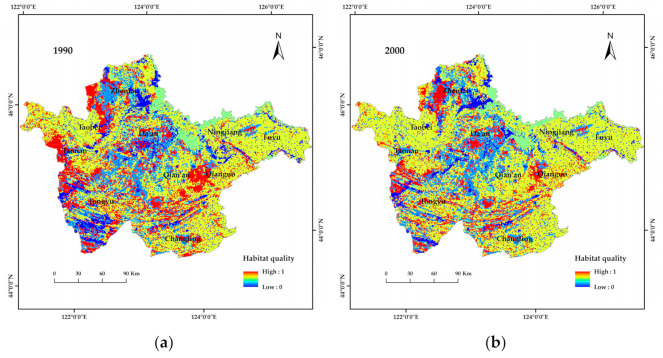

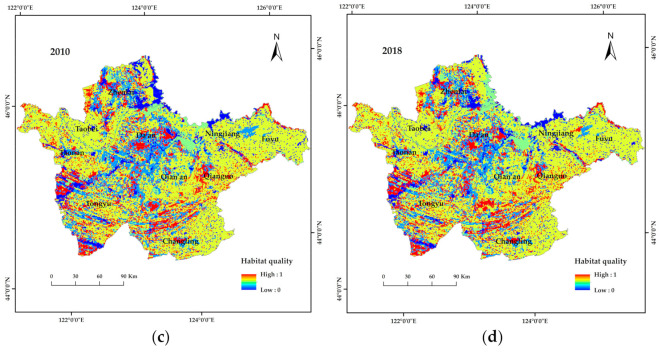
Spatial distribution of habitat quality index values in western Jilin Province from 1990 to 2018. (**a**–**d**), spatial distribution of habitat quality in western Jilin Province in 1990, 2000, 2010 and 2018, respectively.

As illustrated in Figure 9, the habitat quality index of the study area could be spatially characterized as low in the middle region and high in the surrounding areas. This was especially relevant in 1990, as the high-value areas (>0.6641) were mainly distributed in the west of the study area and the junction of Qian’an County and Qianguo County in the east; the main land use types in these regions were grassland and forest land. The high-value areas for habit quality from 1990 had almost disappeared in 2018, whereas the distribution of low-value areas (<0.3320) remained unchanged over the studied period and was mostly concentrated to the middle of the study area, where the main land type was saline-alkali land. From 1990 to 2018, grassland area decreased, but grassland with different coverage levels was always distributed in areas with high habitat quality index values; this indicates that grassland exerts certain maintenance and stabilization effects on habitat quality.

### 3.3. Analysis of Grassland Ecosystem Service Hotspots

#### 3.3.1. Analysis of Ecosystem Service Tradeoffs and Synergies

ArcGIS was used to generate a set number of random sampling points within the grassland of the study area in 1990, 2000, 2010 and 2018. Next, the results for the four ecosystem services of carbon storage, soil conservation, water supply and habitat quality across the four time points were superimposed onto the random sampling points. The ecosystem service data of each point in each period were then extracted for correlation analysis. As the data did not demonstrate normal distribution, Spearman’s correlation analysis was used, with the results shown in Table 3. Data that show a significant positive correlation are indicative of a synergistic relationship between two ecosystem services. Data that show a significant negative correlation are indicative of a trade-off between two ecosystem services. In the cases where no significant correlation was found, the two ecosystem services are compatible.

As can be seen from Table 3, water supply services were significantly negatively correlated with carbon storage, soil conservation, and habitat quality services during the period 1990–2018; this was indicative of a trade-off relationship between water supply and the other three services. Carbon storage was significantly positively correlated with habitat quality and soil conservation services; these results demonstrate synergistic relationships. Soil conservation was also significantly positively correlated with carbon storage. The soil conservation services of grassland are very important due to the observed synergistic relationships with carbon storage and habitat quality. The trade-off between water supply and other services confirmed a climatic cause for grassland salinization. The study period showed large variation in rainfall. When combined with poor soil permeability and the topographic conditions of the study area, this means that excessive rainfall would lead to seasonal water accumulation and salt accumulation. However, drought conditions also tend to lead to salinization, especially in a region with a dry climate and sparse vegetation. Therefore, under the controlled water supply conditions, stabilizing the soil conservation function of grassland can improve the carbon storage capacity of soil and protect, and potentially improve, the habitat quality in the study area.

#### 3.3.2. Identification of Ecosystem Service Hotspots

A certain ecosystem can provide different ecosystem services. In this paper, four ecosystem services in the study area were identified based on the trade-offs and synergies between ecosystem services. Regions with ecosystem service values that exceeded the average value were selected as hotspots for ecosystem services. In class 0 hotspots, none of the four ecosystem services exceeded the average values. In class 1 hotspots, only one ecosystem service exceeded average values. In class 2 hotspots, two ecosystem services exceeded average values. In class 3 hotspots, three ecosystem services exceeded average values. In class 4 hotspots, all four assessed ecosystem services exceeded average values. Based on these classification principles and the analysis of data from the randomly generated points, the main service hotspot types for grassland, along with their proportions, were obtained; the results are summarized in Table 4. Grassland mainly included type 1, 2, and 3 service hotspots. Low-cover grassland mainly included type 1 service hotspots, medium-cover grassland mainly included type 2 service hotspots, and high-cover grassland mainly included type 2 and 3 service hotspots.

Based on the information in Table 4, the types of service hotspots of grassland remained relatively stable from 1990 to 2018, with four types (habitat quality-carbon storage, habitat quality-water supply-carbon storage, habitat quality-carbon storage-soil conservation, and hotspots representing all four services) observed for high- and medium-cover grassland. Low-cover grassland provided two types of hotspots, habitat quality and habitat quality-water supply, with the habitat quality-soil conservation hotspots only present in 2010 and 2018. High- and medium-cover grassland showed similar types of service hotspots, but high-cover grassland was more frequently converted to other land use types than medium-cover grassland between 1990 to 2018; medium-cover grassland mainly showed habitat quality-carbon storage hotspots. In terms of hotspot types, grassland only had the highest proportion of habitat quality-water supply-carbon storage hotspots in 2018. The proportions of habitat quality and habitat quality water supply hotspots remained stable throughout the study period, whereas habitat quality soil conservation hotspots appeared in 2010.

**Figure 10:**
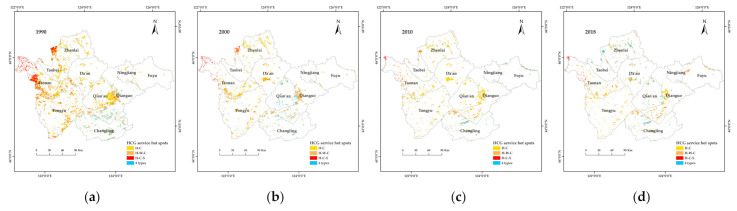

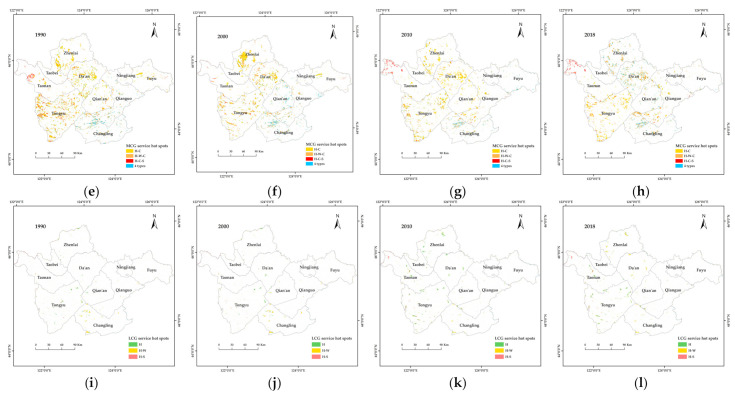
Distribution of ecosystem service hotspot types across grassland with different coverage during 1990–2018. C = Carbon storage; S = Soil conservation; W = Water supply; H = Habitat quality. (**a**–**d**), spatial distribution of service hotspots across high-cover grassland in western Jilin Province in 1990, 2000, 2010 and 2018, respectively; (**e**–**h**), spatial distribution of service hotspots across medium-cover grassland in western Jilin Province in 1990, 2000, 2010 and 2018, respectively; (**i**–**l**), spatial distribution of service hotspots across low-cover grassland in western Jilin Province in 1990, 2000, 2010 and 2018, respectively.

Combining the applied classification of service hotspots and Figure 2 yielded spatial distribution maps for how grassland with different coverage provide service hotspots from 1990 to 2018 (Figure 10). As can be seen in Figure 10, the three service hotspots associated with high-cover grassland decreased year by year and rebounded slightly in 2018. In 1990, the habitat quality-water-supply-carbon storage service hotspots were mainly distributed in the west and south of Qianguo County and the north of Changling County; by 2010, the hotspots west and south of Qianguo County had been transformed into habitat quality-carbon storage service hotspots. In 1990, the habitat quality, carbon storage and soil conservation service hotspots were mainly distributed in the west of Zhenlai County and in the southwest and west of Taonan City. By 2000, the number of these service hotspots had increased in the middle part of Tao Nan City, where the distribution in other areas had not changed; however, the coverage area was only half of what it had been in 1990.

The medium-cover grassland mainly provided two types of service hotspots (habitat quality and habitat quality-carbon storage) across the studied period. In 1990, the habitat quality-carbon storage service hotspots were mainly distributed in Zhenlai County, Da’an City, Tongyu County and to the west of Fuyu City. In 2000, the number of these service hotspots had increased in Zhenlai County, and clusters had formed in the west. However, by 2010, the area of habitat quality-carbon storage hotspots originally distributed in western Zhenlai County had disappeared. In 2018, the habitat quality-carbon storage service hotspots in Da’an City and Zhenlai County were transformed into habitat quality-water supply-carbon storage service hotspots, whereas the habitat quality-carbon stocking-soil conservation service hot spots were transformed into four service hot spots, with the overall service function improved.

In 1990 and 2000, the habitat quality service hotspots were mainly distributed in Tongyu County, and the habitat quality-water supply service hotspots were mainly distributed in Tongyu County and Da’an City. However, due to the small area of low-cover grassland, the ecological quality service hotspots were mainly distributed in Tongyu County and Da’an City. As such, these hotspots not only had scattered spatial distribution, but also small total area. In 2010, the amount of habitat quality service hotspots began to increase, and they were predominantly scattered in the western part of the study area. In 2018, the low-cover grassland hotspots in Zhenlai County and Da’an City changed from habitat quality type to habitat quality-water supply type.

## 4. Discussion

### 4.1. Feasibility of Evaluating Grassland Ecosystem Services Using the InVEST Model

All evaluation models are based on data. When data are difficult to obtain or complicated to process, the model is likely to fail [46,47]. In this case, it is better to choose an evaluation mode that is relatively easy to operate, and which utilizes data that are easy to obtain. Currently, the InVEST model is one of the most mature models for quantitative research on ecosystem services and is able to comprehensively simulate ecosystem services [48,49] from data that are easy to obtain. The western part of Jilin Province is located in the Songnen Plain, which is one of three major saline-alkali regions in the world and a typical ecologically fragile area. Conventional sampling methods usually cause some extent of damage to the regional ecological environment [12], which is not conducive to maintaining regional ecological stability. Therefore, any investigation of the ecosystem services of salinized grassland in western Jilin Province should rely on objective data that are representative of the region. The InVEST model is one of the most suitable models for such assessments because when compared to other ecosystem assessment models such as Esvalue and EcoMetric, it provides obvious advantages in spatial representation, visualization, and application of results [50].

Since 2009, the InVEST model has been widely used [51]. Many scholars have used the InVEST model to evaluate ecosystem services in different regions, and they have obtained results that are consistent with the actual situation [37,38,39,40,41,42]. In this paper, the InVEST model was used to evaluate four ecosystem services (carbon storage, soil conservation, water supply and habitat quality) in the salinized grasslands for western Jilin Province; as a result, four evaluation results from the period between 1990–2018 were obtained. The data required for the assessment represent objective reality, and the assessment process conforms to the InVEST model operating specifications and ecosystem service assessment theory. Therefore, the ecosystem services results are in line with objective reality.

### 4.2. Analysis of the Distribution of Ecosystem Services across Salinized Grassland in Western Jilin Province

As humans, we use the natural environment to obtain the materials we need for survival and continuous development; unfortunately, these activities negatively affect the ecosystem by interfering with natural laws and ultimately leading to noticeable changes in ecosystem services [52,53]. Land quality is a factor that directly affects the quality of ecosystem services.

Based on the quantitative measurement of ecosystem services of salinized grasslands in western Jilin Province using the InVEST model, this paper analyzes the relationship between various ecosystem services and identifies ecosystem service hotspots. The research results show that, between 1990–2018, the ecosystem services in western Jilin Province experienced an overall downward trend, there are trade-offs between certain ecosystem services, and the overall service quality is poor. Correspondingly, the grassland area and coverage decreased. Human factors are the fundamental cause of this situation. The intensification of human activities has increased the conversion of grassland into cultivated land, and land use has become more frequent. Moreover, land protection measures have not been effectively implemented, which has increased soil salinity. A similar result was obtained by Li et al., who reported three major ecological problems for the grasslands of western Jilin Province, namely, grassland degradation, soil salinization and wetland shrinkage; Li et al. also identified human factors as the predominant cause of these problems [54]. Therefore, grassland restoration and soil protection in western Jilin Province should be strengthened to improve the local ecosystem.

Grassland quality directly affects the quality of ecosystem services. As such, degraded grassland yielded low-quality ecosystem services in comparison to higher-quality grassland. Studies have shown that high-quality grassland ecosystem services are about 72.16% higher in quality than what is provided by degraded grassland [3]. Generally, high-quality grasslands are located in a suitable geographical environment and climate. The grassland assessed in the presented research is located in a saline-alkali area, which is characterized by a harsh environment and a typically degraded grassland. The research results show a trade-off relationship between water supply and other ecosystem services, which further confirms that changes in the climate aggravate grassland salinization, resulting in a decrease in grassland quality and a decline in ecosystem services. The study area showed significant variation in rainfall and poor soil permeability. Furthermore, the topographic conditions of the study area will lead to the seasonal accumulation of water and salt, with the salt concentration of the soil increasing after drying and potentially causing secondary hydrosalinization. However, too little rainfall can also lead to salinization in a region with a dry climate and sparse vegetation. Therefore, proper control of the water supply is a key approach to mitigating further grassland salinization.

### 4.3. Practical Applications of the Research Results

The presented research, which investigated grassland cover changes and ecosystem services in western Jilin Province, as well as identified temporal and spatial changes in grassland ecological service hotspots, provides a basis for decision-making that aims to further strengthen the protection and restoration of grasslands in western Jilin Province. The core ecological services and ecological functions of grassland in western Jilin Province are windbreak and sand fixation, along with soil conservation. However, increasing salinization of the area in recent years has caused the continuous degradation of grassland area and the gradual decline in function, which seriously threatens the ecology and economic development of the region. The results of this paper identify two main reasons for the deterioration of grassland ecosystem services in western Jilin Province. One is interference by humans, notably the intensification of agricultural production and urban expansion, both of which require the conversion of grassland to cultivated land and result in grassland degradation. The second cause is changes in the climate. Imbalances in water supply have resulted in the accumulation of saline-alkali components in the topsoil of the grassland, which has aggravated salinization in a large area of the grassland in the study area. When grassland salinization reaches a certain level, grassland degradation will become even more severe. Therefore, it is necessary to strengthen the protection of the grasslands in western Jilin Province and proactively return farmland to grassland in the region. Secondly, the region should increase the artificial regulation of water supply to alleviate increasingly serious salinity in western Jilin Province. This is not relevant to the ecological functioning of the study area, but is in line with the guidelines for strengthening ecological protection and restoring grasslands set forth in the “Implementation Plan for Accelerating the Construction of Ecological Civilization” issued by Jilin Province in 2016.

### 4.4. Deviation Analysis and Research Prospects

In this paper, four grassland ecosystem services in western Jilin Province were studied using the InVEST model. Spearman’s correlation analysis was used to explore the trade-offs and synergies among these services, after which ecosystem service hotspots were identified. However, the complexity of the research problem and the limitations of the method mean that further investigation of the topic is needed.

Since the interpreted remote sensing images were cloud-free images from mid-June to late September in Northeast China, the indicators of carbon storage, soil conservation and water supply ecosystem services of cultivated land were higher than what was observed for other land types. It is important to note that these analyses do not reflect the situation of ecosystem services for the entire year. However, the vector data of land use types are derived from the Data Center of Resources and Environmental Sciences of the Chinese Academy of Sciences, which ensures the accuracy of interpretation and that an adequate amount of data was analyzed. Furthermore, the data analyzed for each time period (1990, 2000, 2010 and 2018) represented the same quarter in the year, so the comparisons between time points can be deemed reliable. Secondly, the presented research analyzed the temporal and spatial variation in ecosystem services, as well as their relationships, and discussed the possible factors that affect the measured ecosystem services. This represents an initial analysis of the situation, and as such, future research should focus on the fundamental driving factors for changes in the ecosystem services of salinized grassland in western Jilin Province.

## 5. Conclusions

The threat of grassland degradation in western Jilin Province is increasing year by year and has become the main problem facing the ecological environment in this region. The main reason for the sharp decrease in grassland area from 1990 to 2018 has been the conversion of grassland into cultivated land, followed by the degradation of grassland into saline-alkali land. The downgrading and transformation of grassland coverage is also an important manifestation of grassland degradation.

From 1990 to 2018, downward trends were observed for the carbon storage, soil retention and water supply of grasslands in western Jilin Province, whereas habitat quality remained basically stable. The total amount of grassland, as well as its coverage, are intricately linked to the provided ecosystem services, and maintaining the stability of ecosystem services is crucial to future efforts to increase grassland area and coverage.

A trade-off relationship was identified for water supply and other assessed services (e.g., carbon storage, soil retention, and habitat quality). This indirectly confirms a climatic cause for grassland salinization in western Jilin Province, with precipitation acting as the main influencing factor. Various types of ecosystem service hotspots were identified for the investigated grassland, but there were scarce hotspots that offered all four types of ecosystem services; this indicates that the grassland in western Jilin Province is of low quality from an ecosystem standpoint.

Undisturbed grassland salinization exerts a negative effect on the ecosystem, resulting in decreased vegetation coverage, increased desertification, and ecosystem degradation. This will affect the relationships between ecosystem services, which can be seen in changes in various ecosystem characteristics, e.g., water supply, soil conservation service, carbon storage and habitat quality, over a period of nearly 30 years.

## Figures and Tables

**Table 1:** The contribution rates of conversion-in and conversion-out for land use types during 1990–2018 (%).

Time Period	Contribution Rate	CL ^1^	FL ^2^	WL ^3^	URL ^4^	UL ^5^	HCG ^6^	MCG ^7^	LCG ^8^	SAL ^9^
1990–2000	Conversion-in	51.56	15.65	1.64	0.35	7.16	3.27	6.68	0.28	13.41
	Conversion-out	10.69	3.75	7.86	0.00	17.61	36.25	15.92	0.33	7.58
2000–2010	Conversion-in	29.46	9.36	4.50	5.07	14.65	8.29	11.16	3.30	14.21
	Conversion-out	22.12	6.94	13.39	3.39	10.12	14.77	14.19	1.42	13.64
2010–2018	Conversion-in	35.36	11.80	18.01	5.82	7.30	5.03	12.12	2.00	2.58
	Conversion-out	17.58	8.74	6.13	0.55	26.91	7.44	5.92	0.25	26.48
1990–2018	Conversion-in	44.61	12.74	3.66	3.84	7.64	5.00	8.05	2.20	12.26
	Conversion-out	13.30	3.89	9.65	1.71	15.25	28.20	13.83	0.89	13.29

^1^ Cultivated land; ^2^ Forest land; ^3^ Water land; ^4^ Urban and rural construction land; ^5^ Unused land; ^6^ High-cover grassland; ^7^ Medium-cover grassland; ^8^ Low-cover grassland; ^9^ Saline-alkali land.

**Table 2:** Habitat quality index changes of different land use types from 1990 to 2018.

Land Type	1990	2000	2010	2018	Average
CL ^1^	0.5998	0.5999	0.5999	0.5999	0.5999
FL ^2^	0.9782	0.9796	0.9785	0.9776	0.9785
WL ^3^	0.4894	0.4882	0.4844	0.4850	0.4867
URL ^4^	0	0	0	0	0
UL ^5^	0	0	0	0	0
HCG ^6^	0.9850	0.9806	0.9804	0.9793	0.9814
MCG ^7^	0.9845	0.9840	0.9816	0.9817	0.9830
LCG ^8^	0.9881	0.9855	0.9828	0.9842	0.9851
SAL ^9^	0.1997	0.1996	0.1996	0.1996	0.1996
Average	0.5805	0.5797	0.5786	0.5786	--

^1^ Cultivated land; ^2^ Forest land; ^3^ Water land; ^4^ Urban and rural construction land; ^5^ Unused land; ^6^ High-cover grassland; ^7^ Medium-cover grassland; ^8^ Low-cover grassland; ^9^ Saline-alkali land.

**Table 3:** Correlation of grassland ecosystem services between 1990 and 2018.

1990	Carbon Storage	Soil Conservation	Water Supply	Habitat Quality
Carbon storage	1.000	0.276 **	−0.454 **	0.811 **
Soil conservation		1.000	−0.242 **	0.221 **
Water supply			1.000	−0.498 **
Habitat quality				1.000
2000	Carbon storage	Soil conservation	Water supply	Habitat quality
Carbon storage	1.000	0.311 **	−0.414 **	0.811 **
Soil conservation		1.000	−0.197 **	0.236 **
Water supply			1.000	−0.403 **
Habitat quality				1.000
2010	Carbon storage	Soil conservation	Water supply	Habitat quality
Carbon storage	1.000	0.330 **	−0.468 **	0.790 **
Soil conservation		1.000	−0.136 **	0.241 **
Water supply			1.000	−0.502 **
Habitat quality				1.000
2018	Carbon storage	Soil conservation	Water supply	Habitat quality
Carbon storage	1.000	0.316 **	−0.182 **	0.779 **
Soil conservation		1.000	−0.060 **	0.232 **
Water supply			1.000	−0.205 **
Habitat quality				1.000

Notes: ** indicates that the correlation was significant at the 0.01 level (two-tailed).

**Table 4:** Types and proportions of main service hotspots in grassland with different coverage during 1990–2018.

Land Type	1990	Proportion	2000	Proportion	2010	Proportion	2018	Proportion
HCG	H-C	33.54%	H-C	23.69%	H-C	29.93%	H-C	14.74%
	H-W-C	9.51%	H-W-C	13.32%	H-W-C	5.67%	H-W-C	16.79%
	H-C-S	15.07%	H-C-S	8.87%	H-C-S	6.72%	H-C-S	4.84%
	4 types	3.03%	4 types	3.94%	4 types	2.30%	4 types	5.51%
MCG	H-C	23.93%	H-C	28.45%	H-C	34.85%	H-C	18.73%
	H-W-C	4.84%	H-W-C	8.54%	H-W-C	4.59%	H-W-C	20.90%
	H-C-S	5.81%	H-C-S	5.63%	H-C-S	6.57%	H-C-S	4.98%
	4 types	1.98%	4 types	3.88%	4 types	1.98%	4 types	5.22%
LCG	H	1.04%	H	1.50%	H	5.39%	H	4.33%
	H-W	0.99%	H-W	1.77%	H-W	0.91%	H-W	2.87%
					H-S	1.04%	H-S	0.82%

Notes: C = Carbon storage; S = Soil conservation; W = Water supply; H = Habitat quality; 4 types refer to hotspots that provide all four ecosystem services at levels higher than the average value (Carbon storage, soil conservation, water supply, and habitat quality); HCG = High-cover grassland; MCG = Medium-cover grassland; LCG = Low-cover grassland.

## Data Availability

The data presented in this study are available in the article.
